# The complete mitochondrial genome sequence and gene organization of *Istigobius campbelli* (Perciformes, Gobiidae) with phylogenetic consideration

**DOI:** 10.1080/23802359.2019.1598809

**Published:** 2019-10-01

**Authors:** Linzi Zhang, Chunyan Ma, Ming Zhao, Fengying Zhang, Yamei Wang, Lingbo Ma

**Affiliations:** a Key Laboratory of East China Sea and Oceanic Fishery Resources Exploitation, Ministry of Agriculture, East China Sea Fisheries Research Institute, Chinese Academy of Fishery Sciences, Shanghai, China;; b College of Fisheries and Life Sciences, Shanghai Ocean University, Shanghai, China

**Keywords:** *Istigobius campbelli*, mitochondrial genome, phylogenetic tree, evolutionary relationships

## Abstract

The complete mitochondrial genome DNA sequence of *Istigobius campbelli* was 16,527 bp in length. It consists of 13 protein-coding genes, two ribosomal RNA genes, 22 transfer RNA genes, and 1 control region. 28 of the 37 genes were encoded on the heavy strand, and 9 of them were encoded on the light strand. The overall base composition of the genome is 27.84% A, 25.81% T, 29.68% C, and 16.67% G. The phylogenetic tree suggested *I. campbelli* was genetically closest to *Acentrogobius pflaumii* and *Oxyurichthys formosanus*. This study could provide valuable information for further studies on *I. campbelli*.

*Istigobius campbelli*, a member of Gobiidae is broadly distributed in Northwest Pacific: southern Japan, Taiwan, Hong Kong and Common in sandy bottoms of shallow waters. Moreover, they were observed living solitarily or in small groups near crevices or under stones (Blaber 1980). The maximum length of the male is 7.1 cm and female is 7.8 cm (Murdy and Hoese 1985). Mitochondrial DNA plays a significant part on the studies of population genetics, phylogenetics, and evolution (Avise et al. 1984; Zhong et al. 2013; Xia et al. 2015). So far, there was no introduction about the complete mitochondrial genome of *I. campbelli.* The study is important for further research on genetics and evolution of *I. campbelli*.

The specimen of *I. campbelli* was collected from Fuqing, Fujian, China (25°50′2″N, 119°27′35″E) in April 2017. it was stored in East China Sea Fisheries Research Institute, Chinese Academy of Fishery Science. Genomic DNA was extracted from muscle tissue using Animal Genomic DNA Extraction Kit (TIANGEN, Beijing, China) according to the manufacturer’s recommended protocol. In the present study, the complete mitochondrial DNA sequence of *I. campbelli* has been determined by the Roche 454 Genome Sequencer FLX System. The total length was 16,527 bp (GenBank accession no.MK409978). The base composition of its mitogenome is 27.84% for A, 25.81% for T, 29.68% for C, and 16.67% for G. The overall A + T content of the mitochondrial genome is 53.65%. This genome includes 13 protein-coding genes, two ribosomal RNA genes, 22 transfer RNA genes, and 1 control region. Twenty eight of these 37 genes were encoded on the heavy strand, and 9 were encoded on the light strand just as in other teleosts (Song et al. 2016). The overall length of protein-coding genes is 11,426 bp. Two kinds of start codons (ATG and GTG) were identified in 13 protein-coding genes. Eight genes ended with TAA, whereas five genes had incomplete stop codons T. The length of control region (D-loop) which has a higher A + T content (61.23%) is 859 bp, and its overall nucleotide composition is 31.43% for A, 22.47% for C, 16.30% for G, and 29.80% for T.

To assess its phylogeny and evolution, the phylogenetic tree was constructed with significant bootstrap supports based on the Neighbour-joining method in MEGA 7.0 (Figure 1). *Lateolabrax japonicus* and *Micropterus floridanus* were used as an out-group. The NJ tree showed that *I. campbelli* clustered with *Acentrogobius pflaumii* and *Oxyurichthys formosanus*, then together with other two species in genus *Glossogobius* and two species in genus Bathygobius in this study, further together with some other species in family Gobiidae forming a big branch. This study will be important to the genetic conservation and the phylogenetic classification of *I. campbelli*.

**Figure 1.**
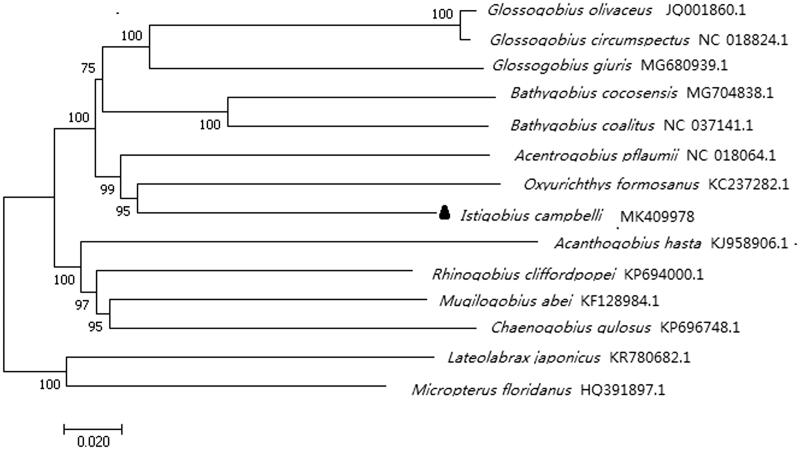
phylogenetic position of *Istigobius campbelli* within Perciformes based on 12 protein-coding genes (without ND6) using neighbour-joining method. *Istigobius campbelli* is highlighted with a black triangle.
